# Community Engagement Before Initiation of Typhoid Conjugate Vaccine Trial in Schools in Two Urban Townships in Blantyre, Malawi: Experience and Lessons

**DOI:** 10.1093/cid/ciy1110

**Published:** 2019-03-07

**Authors:** James E Meiring, Rodrick Sambakunsi, Elvis Moyo, Theresa Misiri, Felistas Mwakiseghile, Pratiksha Patel, Priyanka Patel, John Ndaferankhande, Matthew Laurens, Kate Gooding, Melita A Gordon

**Affiliations:** 1 Malawi-Liverpool Wellcome Trust Clinical Research Programme, Blantyre, Malawi; 2 Oxford Vaccine Group, Department of Paediatrics, Oxford University, United Kingdom; 3 Center for Vaccine Development and Global Health at the University of Maryland School of Medicine, Baltimore, MD; 4 Institute of Infection and Global Health, University of Liverpool, United Kingdom

**Keywords:** typhoid, typhoid conjugate vaccine, Africa, community engagement, vaccine acceptability

## Abstract

**Background:**

To determine the efficacy of a new typhoid conjugate vaccine in an endemic setting in sub-Saharan Africa, the Typhoid Vaccine Acceleration Consortium is conducting a phase-3 randomized controlled trial in Blantyre, Malawi. This article describes community and stakeholder engagement activities before and during the trial, challenges, and lessons learned.

**Methods:**

In October 2017, Malawi-Liverpool Wellcome Trust (MLW) organized a wide range of community engagement activities, including meetings with Ministry of Health and Education officials at the district and facility level, local community leadership, and parent teacher association groups. We engaged media outlets to include local and international television, radio, and print media. Community members were informed directly through a study jingle played via loudspeaker from a van and by community-based activities.

To review engagement activity effectiveness: The MLW team met to discuss progress and challenges; and a focus group discussion (FGD), consisting of trial staff, sought feedback from the community on each engagement modality.

**Results:**

The school-based vaccine campaign increased community participation exceeding recruitment targets to date (on average, >200 children/day).

**Conclusions:**

The FGD concluded that the van and local activities improved awareness and turnout for the trial, but prior engagement with local government and community leadership is an essential mechanism to provide details of the study, answer questions, communicate the value of the study, and address safety concerns. Effective community engagement is essential in a large intervention trial. Multiple channels of communication are required to reach the community and deliver information needed for participation and provide opportunity for dialogue with the trial team.

Typhoid fever is a major cause of febrile illness in many low- and middle-income countries, responsible for an estimated 12 million infections globally each year and over 128 000 deaths [1–4]. Disease burden is borne mainly by infants and school-age children [5]. Cases increased markedly throughout east and southern Africa after a new multidrug-resistant (MDR) strain appeared [6–8]. Although disease rates decreased in developed countries with improvements in water and sanitation, the threat of escalating antimicrobial resistance (AMR) could reverse these trends [9, 10]. Therefore, prequalification of a new typhoid conjugate vaccine (Vi-TCV), Typbar-TCV®, by the World Health Organization (WHO) [11] after promising efficacy results from the Oxford human challenge model [12] is an encouraging development, meaning control of the global typhoid burden may be possible through vaccination.

After WHO prequalification of Typbar-TCV®, the WHO Strategic Advisory Group of Experts on Immunization recommended Vi-TCV in countries with elevated burden of disease or increased rates of AMR [13]. Through funding from the Bill & Melinda Gates Foundation, the Typhoid Vaccine Acceleration Consortium (TyVAC) was formed to accelerate introduction of typhoid conjugate vaccines (TCVs) into low- and middle-income countries [14, 15] and provide evidence for vaccine impact through a series of efficacy trials in endemic countries.

Blantyre, Malawi has documented increased typhoid cases isolated from the central tertiary care hospital after a new MDR strain emerged [6, 8, 16]. The Strategic Typhoid Alliance across Africa and Asia (STRATAA), a collaboration between the University of Oxford, the Malawi-Liverpool Wellcome Trust (MLW), and other partners has further characterized elevated disease incidence using community-based typhoid epidemiology surveillance in Ndirande township, Blantyre [17].

The TyVAC Malawi trial is a phase-3 randomized, double blind, controlled trial of the clinical efficacy of TCV in 28 000 children aged 9 months through 12 years. Children are randomized 1:1 to receive Vi-TCV or a meningitis A conjugate vaccine. TyVAC Malawi will follow participants, through passive surveillance for febrile illness, up to 3 years. The primary outcome is blood culture-confirmed *Salmonella* Typhi (*S*. Typhi) infection.

Community stakeholder engagement is increasingly recognized as essential for both ethical research practice and trial feasibility. Engagement can build trust with communities, generate awareness and understanding of the research, promote ownership among local stakeholders, and seek feedback that helps researchers ensure trial procedures are appropriate in the local setting. Engagement thus has the potential to reduce exploitation and harm, support recruitment [18–20], facilitate research activities and enable the feedback of findings to the population; however, community engagement is a complex undertaking, and there are few published examples to provide lessons for future practice. Previous research in Malawi has described varied community perceptions of vaccine trials [21], the importance of community engagement [22], and challenges surrounding engagement strategies [23]. This article describes the largest community and stakeholder engagement effort undertaken by MLW and draws on reflections from trial staff to evaluate the relative success of different engagement modalities.

## METHODS

### Setting

**Figure 1.**
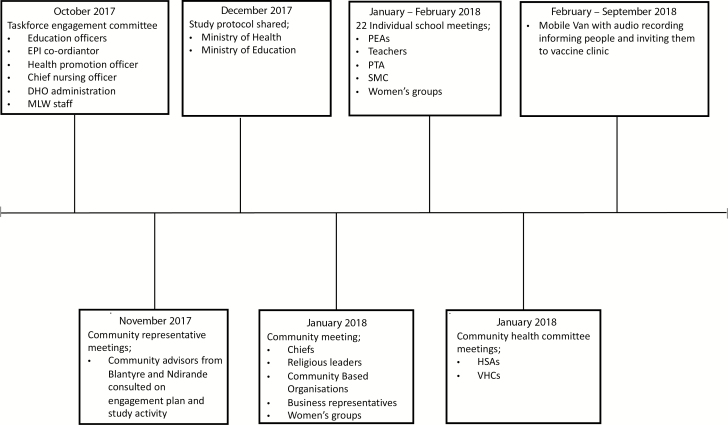
Timeline of engagement activities. Abbreviations: DHO, District Health Officer; EPI, extended programme of immunization; HSA, health surveillance assistants; MLW, Malawi Liverpool Wellcome Trust; PEA, primary education advisors; PTA, parent teacher association; SMC, school management committee; VHC, village health committee.

**Figure 2.**
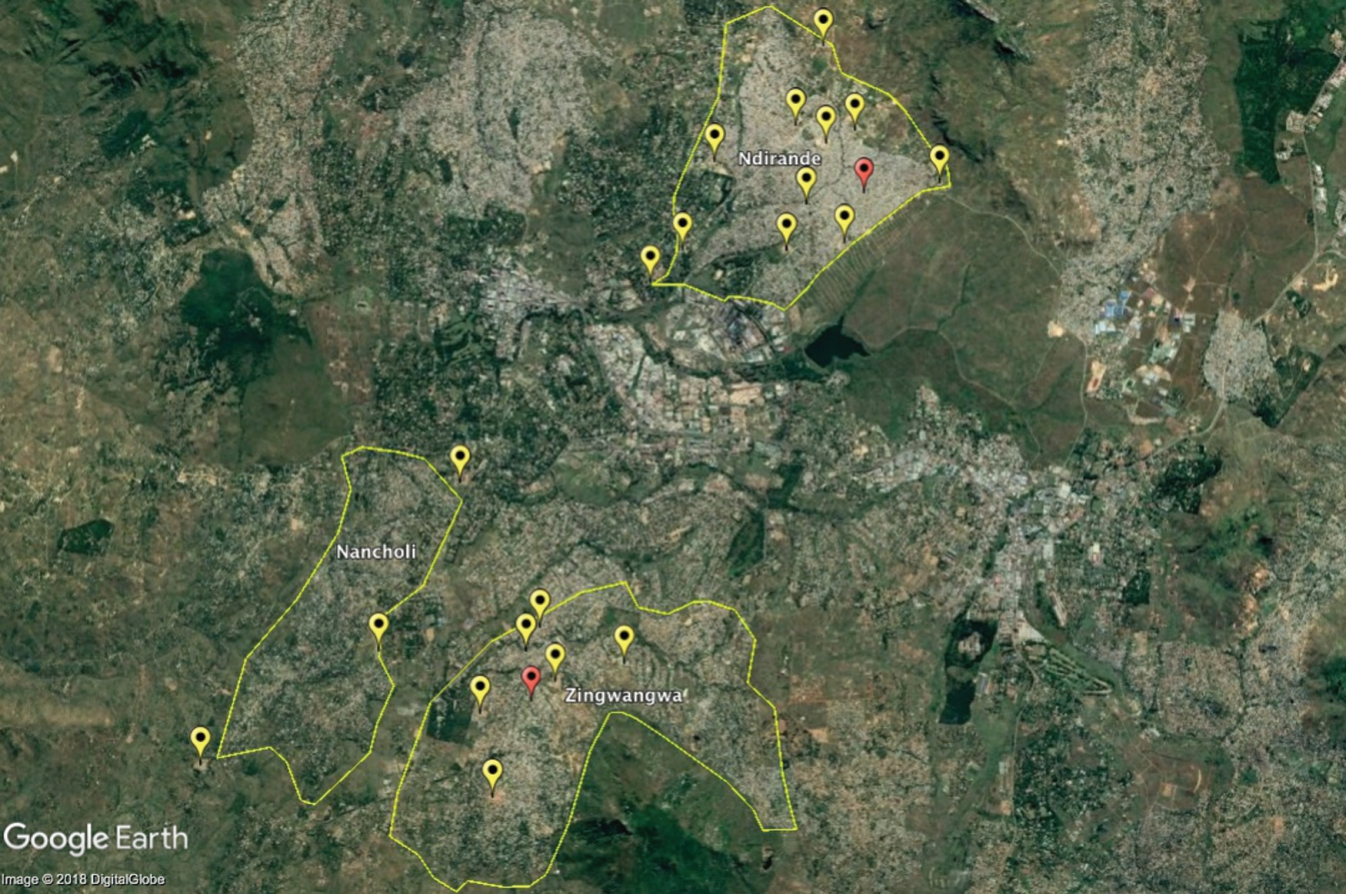
Blantyre city with Ndirande (upper middle), Zingwangwa (lower middle), and Nancholi (lower left) townships with schools (yellow), and health centers(red) located. Source: Google Earth.

In October 2017, approximately 5 months before the first vaccination, MLW conducted a range of engagement activities within the communities of Ndirande and Zingwangwa townships (Figure 1). Both townships are densely populated urban centers with a combined population of roughly 200 000 people. For sub-Saharan Africa, they are a typically youthful population [24] with increased rates of in-migration as people relocate from villages for economic and educational opportunities. Vaccination administration occurred at 2 health centers and multiple primary schools throughout the community (Figure 2).

### Community Engagement Activities

- Community engagement taskforce
  Under the guidance and leadership of the Blantyre district health office (DHO), the taskforce consisted of education officers, school health and nutrition officers, extended programme immunization (EPI) officers, health promotion officers, the chief nursing officer, members of the Malawi TyVAC team, and DHO administration. The taskforce designed and implemented engagement activities for trial initiation. The DHO provided district-level permission to perform the study and to use designated health and educational facilities for study activities. This permission and support were essential to gain facility access and be seen as a legitimate activity by the community. The taskforce involved 20 people and cost approximately $275 USD.
- Community advisory group (CAG) meetings
  MLW’s resident group of about 20 community advisors consult on study conduct in and around Blantyre. The TyVAC team explained the trial and engagement plan and sought feedback.
- Ministry of Health and Ministry of Education (MoH/MoE)
  The MoH and MoE has to grant permission for study implementation in Blantyre. The Malawi TyVAC trial team provided study protocols to the ministries, answered questions, and clarified planned activities. Once permission was obtained from MoH, management and clinic staff in individual health facilities were trained on the protocol through collaboration with the DHO. Similarly, permission was sought from MoE. Because this was the first time that schools were used for a large-scale medical research trial in Malawi, and because the MoE is less connected with the national research ethics regulatory bodies, TyVAC Malawi obtained face-to-face permission from the Minister and Permanent Secretary for Education prior to regional and local engagement. Subsequent contact with individual schools was mediated by the School Health and Nutrition Officer, who acted as the key link between health and education departments. The support of these 2 ministries was vital for individual school engagement. Study information was disseminated at each school via presentations, discussion groups, flyers, posters, and letters home.
- Community leaders meetings in Ndirande and Zingwangwa
  Over 2 days, TyVAC Malawi brought together traditional village chiefs and headman, ward councillors, religious leaders, business representatives, women’s groups, and community-based organizations to explain the study and answer questions. Approximately 160 community leaders were engaged in this process, costing $840 USD.
- School-based meetings
  The engagement taskforce visited 22 schools to present a study overview and seek participation to establish vaccine clinics on school property. The individual meetings involved primary education advisors, teachers, parent-teacher association, school management committee members, and women’s groups. The taskforce sought permission to recruit within the school, provided clarification, and responded to questions from participants. The schools became the primary location of vaccination for both school aged children and nonschool aged children as mothers often brought multiple children for vaccination. Over 200 people participated in these meetings costing approximately $8250 USD.
- Community health committee meetings
  Health surveillance assistants (HSAs) and village health committees are vital members of the primary health care system in Malawi. These individuals are responsible for immunizations, conducting household follow-up, and distributing health messages to the local community. Engagement with this group focused on their assistance disseminating study information and relaying questions and feedback from the community to the study team. Time was spent ensuring HSAs understood the study protocol, eligibility criteria, and key messages. Approximately 50 people were involved in these meetings at a cost of $1375 USD.
- Mobile van with invitation to come for vaccination
  A local musician created a jingle containing study messages and invited guardians to bring children for vaccination. This jingle played via a mobile van with large speakers on the roof that drove through the communities. The van operated in the early evening when parents were home and before nighttime when vehicle security became problematic. The van campaign began 2 weeks before first vaccinations and continued throughout the vaccination period. Production costs and van use totaled approximately $1925 USD.
- Assessment methods
  To obtain preliminary feedback on the benefits and challenges of different engagement activities, we held reflection meetings with trial and engagement staff throughout the vaccine campaign. To generate additional feedback, we held a focus discussion group with frontline trial staff to better understand community reactions and recruitment challenges. Frontline staff bring particular insight to discussions of community engagement and research ethics because of direct contact with community members and participants [25]. The focus group included 6 frontline trial staff (including fieldworkers and research nurses responsible for approaching community members, meeting community members at trial sites, taking consent, and facilitating vaccination). To support open discussion, a nontrial team member facilitated the 1-hour focus group. All participating staff provided verbal consent to use focus group material within this article.

## RESULTS

**Figure 3.**
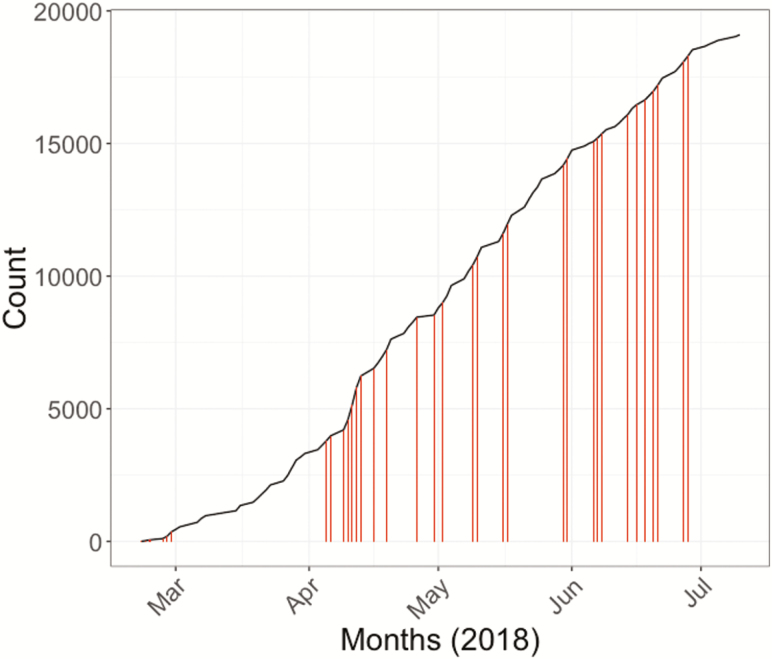
Vaccine recruitment by month. Van sensitization events are shown as bars.

**Figure 4.**
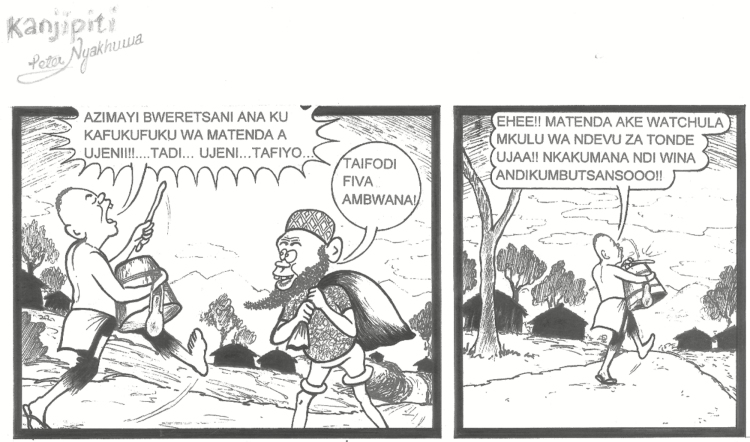
*Kanjipiti Times* Malawi national newspaper cartoon. Translation: “Mothers, bring your children for research study of …..whatchamacallit….Tadi..…ugghhh…..Tafiyo” “Typhoid fever My brother!” “Yes! The disease is the one mentioned by that goat like bearded man! When I meet another person they will remind me again.” The dialogue indicates that the local community know the message of the trial so well that they could remind and correct the village headmen’s drum-beating crier/messenger (who is the titular comic character of the cartoon strip, well-meaning and enthusiastic but not very well educated), when he forgot the details. Credit to Peter Nyakhuwa.

Community engagement was a benefit to the trial and encouraged community uptake in a number of ways:

1. Providing feedback that helped design engagement activities and consent procedures
  Discussions with the CAG and other stakeholders helped the trial team identify appropriate ways to engage the Blantyre community and select specific activities.
2. Creating awareness and increasing recruitment
  Initial consultation with the CAG highlighted areas to focus. A member advised:
  “The use of a mobile van with key messages would be effective in recruitment for the study. This is still a popular medium by which health, political, and commercial messages are delivered to mass audiences in Blantyre and TyVAC should take advantage of this.”
  The mobile van increased recruitment success (Figure 3) and as noted by a frontline trial team member:
  “The van is best. Mostly they do it at night when everyone is almost home, and it was very effective. Almost everyone got the information. Everyone was saying ‘during the night they were saying this, they were saying this, we have to go there.’ When the van has gone around, we do have good turn up.”
  The community engagement resulted in a cartoon appearing in a major newspaper. The appearance of this cartoon, unsolicited and entirely unrelated to the public engagement teams, indicated engagement penetration with the general public. When the cartoonist was contacted, he confirmed that the study van campaign and other engagement efforts prompted him to write the cartoon (Figure 4).
3. Opportunity to provide information and answer questions
  Although the van increased awareness of the study, which helped recruitment, other channels provided opportunities for the community to ask questions. A CAG member advised:
  “Engagement of religious leaders within the community would be important as many parishioners continue to seek advice on health and study enrolment from these leaders.”
  Meetings with key stakeholders provided a venue for community members to ask questions and gain understanding of the trial. One vaccinator mentioned:
  “The van will just call them that ‘there is a vaccine.’ But for them to accept it, there should be meetings with the core people in the community, because they can’t ask questions on the van but if you meet them they will be able to ask the questions ‘what is happening.’”
  During the community leadership and school-based meetings, there was a broad range of questions. The dialogue increased the understanding and acceptance of trial activities. (Table 1).
4. Promoting policy interest in future rollout
  The trial and community engagement activities created significant interest in national and international media. The TyVAC Malawi trial team conducted interviews with multiple national newspapers, radio programs, and television broadcasters [26, 27], further increasing community awareness of the trial and raising the topic and importance of typhoid fever and TCVs. As Malawi and other African countries consider applying for Gavi funding for vaccine introduction, these key messages will ensure discussions are well informed, and TCVs are on the national health agenda.

**Table 1.** Example List of Questions Asked During Community Meetings With Leaders, Parents, and Teachers

Study-specific Questions:
• What will happen to individuals recruited to the study but relocate?
• Will participants receive special/extra provisions if they visit health centers with a medical problem?
• Within the 28 000 children to be vaccinated, were specific age categories or groups targeted?
• How long would the immunogenicity study be conducted, and what is involved?
General public health questions on typhoid transmission and control and sanitation and hygiene:
• What is the safety profile of the vaccine, and what effect will it have on the children who receive it?
• Has the vaccine been “certified”?
• Will blood samples be collected in the study?
• How will the process of randomization and blinding/unblinding be performed? How will the study team know which vaccine each child has received?
• Why are only children aged 9 months through 12 years being vaccinated?
• What are the main side effects of the vaccine?
• How will the study enroll children under 5 given they are not in school?
• What is the difference between the 2 vaccines administered?

## LESSONS

1. The value of a combination of activities
  Delivery of messages at a number of levels in the community from grass roots to upper ministry, ensured individuals were aware of the trial and informed of details.
2. Using trusted information channels
  Local leaders provided a channel to disseminate information and answer questions. Community trust in, and accessibility to, local leaders helped reassure the public.
  “In one of the schools the headmistress was saying that some of the parents, they come to confirm – ‘we heard this from our kids, we had letters, is it okay to bring our kids here to be vaccinated?’ Because they trust the teachers, because they are the ones who are with their kids most of the times. So, they went there for confirmation.” (Frontline trial staff, focus group discussion [FGD])
3. The importance of the trial team addressing misconceptions at enrollment
  Despite community engagement activities and community leaders sharing information and answering questions, there were still misconceptions within the community. Providing information and answering questions at enrolment was important for addressing misconceptions and helping individuals to make informed decisions. Information provided at enrollment travels through the wider community as those messages were delivered back to friends and family by participants:
  “One man was told that what they [MLW] are doing with this vaccine might affect fertility; ‘they are doing this so that we should not have children,’ but after explaining to them they said that ‘okay, now we have the right information, we’ll bring some more children.’” (Frontline trial team member, FGD)
4. Selecting appropriate channels for the context
  Although a van with a loudspeaker is a relatively unsophisticated approach compared to social media campaigns and other activities employed by trial teams in other locations, it proved effective in raising awareness and suited the context of low-income, densely populated urban areas. Vans with loudspeakers are commonly used in the local community for advertising and other health campaigns, which meant the community was familiar with the approach.
  Other channels proved less effective in Blantyre. Engagement with local chiefs is a common community engagement strategy but can be problematic [22]. For this trial, chiefs played a limited role because of challenges to hold community meetings in an urban setting where people are occupied with income-generating activities:
  “Chiefs here in town are very different from those in typical villages… in typical villages they really trust in chiefs and they can get information from the chiefs, but here in town it is so hard to call for a meeting. The turnup won’t be all that good. Most people will say; ‘we are busy, we are busy.’” (Frontline trial team member, FGD)
5. The influence of wider social context on community enrollment
  As well as influencing appropriate engagement activities, the social context affected trial uptake and may have encouraged enrolment regardless of engagement activities. In particular, increased vaccine uptake exists in Malawi, with trust in the effectiveness of vaccination [28]. Combined with concern and knowledge about the severity of typhoid, this trust in vaccination encouraged trial participation:
  “Most of them know how terrible typhoid is, like in areas where they are doing the study when they hear that we are vaccinating against typhoid, so some of them have relatives or children who have suffered from typhoid so they said; ‘I have to go.’”
  Personal experience and an awareness of typhoid severity maybe the result of several years of typhoid research and engagement in the community prior to this trial taking place, including door-to-door surveys and health center recruitment [29, 30]. A comment from a community member on social media referencing earlier MLW work highlighted:
  “The [vaccine campaign] has been done after an earlier survey in which results from blood samples taken showed there are active patients with typhoid and they don’t know it.”
  Other aspects of social context included reduced engagement of relatively wealthy families that prefer to attend private sector health facilities covered through health insurance schemes, rather than the government facilities where vaccine administration and follow-up visits occurred. This discouraged attendance in Zingwangwa, a wealthier area than Ndirande.
  “In Zingwangwa most of the parents were not attending just because when we tell them that after vaccinating, when the child is sick you have to go to Zingwangwa health center. So, they say we are on MASM [insurance scheme], so we can’t go there…while a lot of parents at Ndirande they go to the health centre, they go to the government facilities.” (Frontline trial staff, FGD)

## DISCUSSION

The TyVAC Malawi trial team demonstrated effective methods for engagement in 2 densely populated urban communities in southern Malawi. Engagement activities proved effective in raising trial awareness and mobilizing community participation, as demonstrated through the current recruitment rate and feedback from frontline vaccine staff. We also used feedback that enabled appropriate engagement activity design.

A range of activities that approach different community stakeholders is essential to successful community engagement. The social context is important to consider as traditional authorities have less influence than in the past, religious leadership remains influential, and online social media use grows. Use of a van to disseminate public messages to these communities was an inexpensive and effective way to reach large numbers of people. We would recommend it in other urban areas in sub-Saharan African.

Many of these lessons are based on the trial team’s perspectives. A more thorough assessment would require feedback from the community on TyVAC Malawi engagement activities, including reasons behind enrollment and people’s understandings and experiences of the trial. We recognize that our engagement focused primarily on informing and consulting. We did engage at a deeper level with some stakeholders, including partnerships with local and national government, for collaborative decision making throughout the trial process.

## CONCLUSIONS

The TyVAC Malawi experience confirms the value of community engagement on successful trial activities and enabling community awareness and recruitment. Based on our experience and in line with others [31, 32], we recommend:

1. Researchers involve a wide range of stakeholders in discussions.
2. Start community engagement early and continue through the project.
3. Adequate resource allocation to support engagement activities.
4. Use of a broad range of complementary engagement activities.

We believe sharing accounts of engagement activities, including benefits and lessons learned, may encourage and support other trial teams in undertaking effective engagement.

## Notes

***Acknowledgments.*** The authors acknowledge the support of Leslie Jamka for her editorial assistance, all the stakeholders mentioned in this article for their collaboration and particularly the children and guardians of Blantyre for their willing participation and engagement.

***Financial support.*** This publication is based on research funded in part by a grant from the Bill & Melinda Gates Foundation (OPP1151153). J. M. reports grants from the Wellcome Trust for the STRATAA study.

***Supplement sponsorship.*** This supplement is sponsored by the Center for Vaccine Development and Global Health (CVD) at the University of Maryland School of Medicine.

***Potential conflicts of interest.*** All authors: No potential conflicts. All authors have submitted the ICMJE Form for Disclosure of Potential Conflicts of Interest. Conflicts that the editors consider relevant to the content of the manuscript have been disclosed.
